# Outcome of neurosurgical patients in surgical intensive care unit of a tertiary care center

**DOI:** 10.12669/pjms.42.1.12624

**Published:** 2026-01

**Authors:** Qirat Siddiqui, Faraz Shafiq

**Affiliations:** 1 Qirat Siddiqui, (FCPS Anesthesia) Senior Registrar, Department of Anesthesiology, Patel Hospital, Karachi Pakistan. Aga Khan University Hospital, Karachi, Pakistan; 2 Faraz Shafiq, (FCPS Anesthesia) Associate Professor, Department of Anesthesiology, Aga Khan University Hospital, Karachi, Pakistan

**Keywords:** Critical Care, Neurosurgical procedures, Tertiary care centers

## Abstract

**Background & Objective:**

The intensive care unit (ICU) management of neurosurgical patients is critical for early detection of complications, optimizing recovery, and improving outcomes. The study aimed to determine the outcomes of neurosurgical patients admitted to ICU.

**Methodology:**

After taking exemption from ethical review committee, study was conducted from November 2020 to May 2023 at ICU of a The Aga Khan University Hospital, Pakistan. All adult neurosurgical patients requiring ICU admission were included. Data including demographic variables, comorbid conditions, reasons for ICU referral, complications and expected outcomes were gathered and was analyzed using R. Studio.

**Results:**

Total 93 neurosurgical patients (mean age of 40 years, 69% males) were analyzed. Most of them were admitted to ICU after supratentorial craniotomy (40%). ICU course for them was associated with neurological (78%), metabolic (78%), respiratory (56%), cardiovascular (38%), and infectious (34%) complications. 50% of patients required tracheostomy. The mean length of stay (LOS) in ICU was 6.99 days with ICU mortality of 14.6%. Significant number of these patients (77%) were transferred from the ICU to ward care. The average LOS in ward was 8.43 days, with ward mortality of seven percent. The average LOS in hospital stay was 15.3 days. Comparison of adverse outcomes revealed, patients admitted from ward tend to have the longest ICU stay, higher frequency of having neurological deficit, seizures and sepsis. While higher chances of unsuccessful extubation and need of tracheostomy in patients presented with head injury.

**Conclusion:**

Despite medical advances, morbidity and mortality remain high in neurosurgical patients. The high incidence of neurological, metabolic and respiratory complications and related outcome particularly patients coming from ward and after trauma needs special consideration.

## INTRODUCTION

Intensive care unit (ICU) plays a critical role in health care delivery system throughout the world, providing early detection of complications, facilitating prompt intervention and optimizing recovery.^1^ This is paramount for high risk neurosurgical patients, where postoperative ICU monitoring is widely considered essential following surgical intervention.^2^ The existing literature suggest around quarter (24.8%) of neurosurgical patients requires postoperative ICU care after neurosurgical intervention.^3^ The reasons behind this is high acuity of surgical intervention, which carries not only a risk of postoperative complications but also adds to morbidity and mortality.^4^ Furthermore, these patients remain highly vulnerable to systemic complications even during the ICU stay.^5^ Current evidence based on expected outcomes is primarily derived by data from developed world where, resources and administrative framework is different from low middle-income countries (LMIC). Consequently, even with in the same ICU setup, reported outcomes between various pathological conditions can vary.^6^ Understanding of disease pattern related disparities and outcomes is essential to enhance care for particular group of patient.^7^ The long term morbidity and mortality which is supposed to be the worst outcome ranges between 27-59% for neurosurgical patients.^8^ The developing local databases is a cornerstone for improving healthcare quality. Its effectiveness in understanding disease etiology, administrative planning, resource allocation and predicting outcomes is well established.^9^

Therefore, this study was conceived to address the critical gap in local data by analyzing outcomes and complications of neurosurgical patients admitted to ICU of a tertiary care hospital in Pakistan. The aim was to establish a database for guiding quality initiatives, developing administrative protocols and to implement evidence-based care bundles.

The primary objective of this study was to determine the outcomes of neurosurgical patient admitted in ICU at our tertiary care hospital.

## METHODOLOGY

The study design was descriptive, case series, conducted at surgical intensive care unit of The Aga Khan University Hospital from November 2020 to May 2023.

### Ethical Approval:

The study protocol was reviewed and granted exemption by Dr. Rehana Siddiqui, Chairperson of Ethics Review Committee (ERC) of The Aga Khan University, Karachi (Reference number: 2020-5399-14467; Date: November 14, 2020). The sampling technique was non probability, consecutive sampling. Sample size calculation was based on one of the study outcomes, that is mortality, which was 24.8% reported for neurosurgical patients.^3^ Ninety-six neurosurgical patients were needed to estimate expected mortality within 8.3% margin of error with 95% confidence interval.


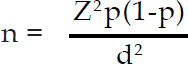


### Inclusion & Exclusion Criteria:

The study included all elective and emergency adult neurosurgical patients aged between 18 to 70 years require further management as a part of their clinical care in ICU. While patients, required readmission to the unit after initial discharge, or left against medical advice were excluded.

The data collection was done prospectively from patient’s confidential medical record, health information management system, daily progress notes, monitoring charts and if needed discharge summaries. Study protocol didn’t interfere in ongoing management plan. One of the primary investigator was responsible for collecting data, which was done on predesigned Performa. The Performa had information regarding demographic characteristics including age, gender and associated comorbid conditions like hypertension (HTN), diabetes mellitus (DM), asthma, ischemic heart disease (IHD), chronic obstructive pulmonary disease (COPD) and chronic kidney disease (CKD). The ICU admission was assessed based on referrals type, which was either from emergency room (ER), operation room (OR) or ward (WR). The reason requiring ICU admission was also recorded. This included low Glasgow coma Scale (GCS), traumatic brain injury (TBI), prolonged surgery, preoperative comorbid condition, new onset of neurological deficit, seizures or bleeding. The number of days patient has been on mechanical ventilation, weaning from ventilator, successful extubation and shifting to WR were also recorded.

The outcomes of these patients were measured in the form of length of stay (LOS) in ICU, and ICU related morbidity, mortality. LOS in ward, ward mortality and overall LOS in hospital. ICU mortality was defined as death happened during ICU stay. It was said to be to “earlier” if occurred within 48 hours, and delayed, if happened after 48 hours of admission. ICU LOS was calculated by calculating the number of days patient spend in unit. “Ward mortality” was defined as death occurred after discharge from ICU during WR stay. Hospital LOS was calculated by number of days patient spend in the hospital after discharged from ICU. ICU related morbidity was estimated in terms of documented systematic complications, occurred during ICU stay. Those complications were predefined, and were recorded as either neurological, cardiovascular, respiratory, renal, metabolic or infectious. However, all the mentioned complications and related documentation was based on progress notes. After discharge from ICU, patients were followed up till discharge to home. The study data was analyzed using R. Studio (version 4.1.2; Boston, MA, USA). Quantitative variables like age, weight, height, BMI, APACHE II score at admission and at 24 hours, and LOS in ICU were reported according to normality assumption. Mean and standard deviation were computed for normally distributed data while median and IQR were for non-normal data. Normality was observed by the Shapiro-wilk normality test and Q-Q plot. Reason of ICU admission, ASA status, gender, comorbid conditions, admission type, morbidity and mortality were reported as frequency and percentage. In univariate analysis, Chi-square test was applied to observe the measure of association between morbidity and mortality with reason of ICU admission. All those variables which had p value < 0.25 were included in multivariate analysis. Stratification analysis was performed to control the effect modifiers like reason of ICU admission, comorbid conditions, morbidity and mortality by using. Chi-square test. Multivariate logistics regression was used to estimate the effect of these variables treated as effect modifiers or confounders and adjusted odd ratio were computed. P≤0.05 was set as threshold.

## RESULTS

The duration of study was 1.5 years. During that period 96 patients were enrolled, out of which, 93 were fulfilling the inclusion criteria. While one patient got bounced back from ward, and two left against medical advice. All of them (n=3) were excluded from final analysis. Mean age of patients was 40 years, out of which 68.8% were males and 31.3% (n=30) were females. Association of comorbid conditions showed predominance of hypertension (33.3%) and diabetes (26%). Most of these patients admitted to ICU from OR (40%). 26% of them came from ER and only 5% were admitted through WR. The admitting diagnosis of these patient was categorized as Supratentorial craniotomy (39.5%,), Traumatic brain injury (TBI) (35.4%), Infratentorial craniotomy (11.4%), and Cerebrovascular disorders (11.4%) and Others (2%). The main reason of ICU admission was postoperative observation (79%) followed by low GCS (73%) and trauma (29%) (Table-I). The mean days patients were on mechanical ventilation were 6.55. Out of them 42.7% of patients were successfully extubated and half of them (50%) required tracheostomy for weaning from mechanical ventilation. For these patients mean LOS in ICU was 6.99 days (Fig.2A). The ICU course for them was associated with significant number of complications. This includes neurological (78%), metabolic (78%) respiratory (56%), cardiovascular (37.5), infectious (34.4%) and renal manifestations (15.6%) (Table-II).

**Table-I:** Demographic Variables

Variables	Total (N=96)
Age Mean (SD)	40.6 (14.8)
Gender	Female (31.3%) Male (68.8%)
BMI Mean (SD)	26.5 (3.88)
***Source of admission***	
OR	40%
ER	26%
WR	5%
***Diagnosis***	
Supratentorial craniotomy	39.5%
Traumatic brain injury	35.4%
Infratentorial craniotomy	11.4%
Cerebrovascular disorders	11.4%
***Reason for ICU admission***	
Postoperative observation	79%
Low GCS	73%
Trauma	29%
Seizures	5%
Bleeding	5%

**Table-II:** Systemic complications during ICU stay.

Systemic complications	Frequency (%)
Neurological	78%
Metabolic	78%
Respiratory	56%
Cardiovascular	37.5%
Infectious	34.4%
Renal	15.6%

**Fig.1:**
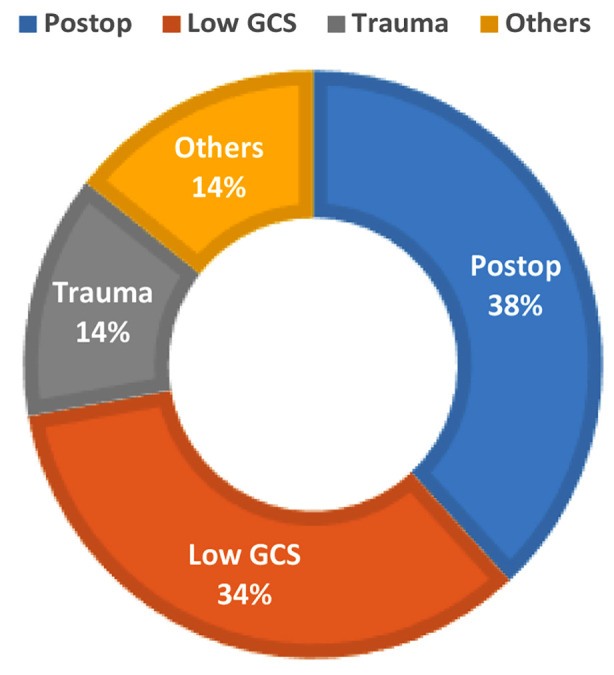
Reasons of ICU Admission.

**Fig.2:**
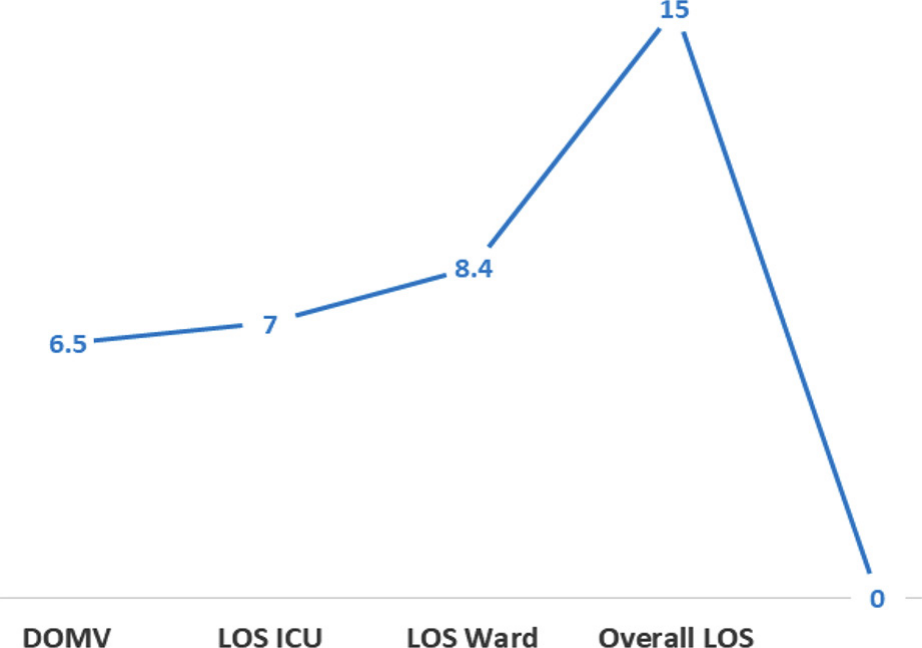
(A and B): Outcome of patients A: Comparison of number of days B: Comparison of Mortality

A: Comparison of number of days


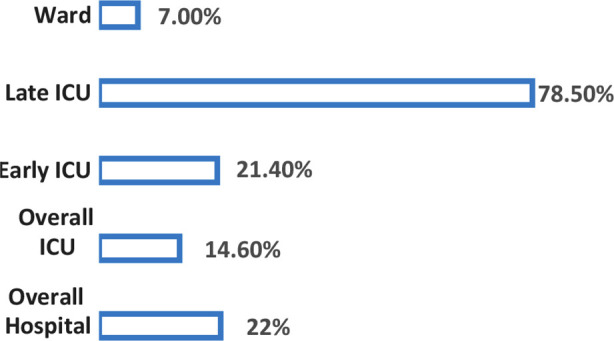


B: Comparison of Mortality.

**Table-III(A):** Comparison of outcomes with Source of admission and Diagnosis.

Outcomes	ER (N=26)	OR (N=65)	WR (N=5)	ST (N=38)	Trauma (N=34)	CV (N=11)	IT (N=11)
Un Successful extubation	57.7%	55.4%	60.0%	52.6%	73.5%	45.5%	27.3%
Need of tracheostomy	57.7%	47.7%	40.0%	44.7%	67.6%	36.4%	27.3%
Withdrawal of care DNAR	11.5	16.9%	20.0%	15.8%	20.6%	9.1%	9.1%
ICU mortality	7.7%	16.9%	20.0%	18.4%	17.6%	0%	9.1%
Mortality in ward	7.7%	5 (7.7%)	0%	2.6%	11.8%	9.1%	9.1%
Stay in ICU Mean (SD)	5.76 (2.91)	7.05 (6.12)	12.4 (8.96)	7.92 (6.92)	7.47 (4.98)	6.20 (4.61)	3.73 (3.20)
Stay in Ward Mean (SD)	8.36 (4.92)	8.73 (10.2)	5.00 (3.74)	8.63 (11.2)	8.09 (7.32)	10.4 (5.08)	7.09 (6.47)
Overall hospital stay. Mean (SD)	13.8 (6.06)	15.7 (10.4)	17.4 (8.23)	16.7 (11.3)	15.3 (8.26)	16.5 (6.17)	10.9 (5.30)

ER: Emergency, OR: Operating room, WR: Ward, ST: Supratentorial craniotomy, CV: Cerebrovascular abnormalities, IT: Infratentorial Craniotomy

P value was not statistically significant for any variable

Overall, 14.6 % of patients in this cohort expired in ICU. Early ICU mortality occurred in 21.4%, while 78.5% died after the 48 hours (Fig.2B). For 15.6% of patients, do not resuscitate code was decided considering their disease course. Significant portion of these patients (77%) were transferred from the ICU to ward care. The mean LOS in ward was 8.43 days, with a ward mortality rate of 7%. The overall mean LOS in hospital stay was 15.3 days. Comparison of adverse outcomes and complications with source of admission and diagnosis (Table-III A, B) revealed patients admitted from the WR tend to have the longest ICU stay of 8.9 days (p value 0.056), higher frequency of having new onset on neurological deficit, seizures and sepsis (P values 0.024, 0,017, 0.039 respectively). Patients with TBI had higher chances of unsuccessful extubation (73. 5%) and need of tracheostomy (67.6%). P value 0.061. Similarly, ICU mortality and overall in hospital mortality was higher but statistically insignificant in patients having trauma.

**Table-III(B):** Comparison of Systemic Complications with Source of admission and Diagnosis.

Systemic Complications	ER (N=26)	OR (N=65)	WR (N=5)	ST (N=38)	Trauma (N=34)	CV (N=11)	IT (N=11)
Neurological	46.2%	63%	140%	100%	82.4%	27.3%	36.4%
Cardiovascular	23.1%	43%	40%	47.3%	88.4%	0%	36.4%
Respiratory	61.5%	52.4%	80%	54.8%	76.5%	27.3%	63.7%
Metabolic	80.7%	75.4%	100%	26.3%	82.3%	72.7%	9.1%
Renal	11.5%	15.4%	40%	18.4%	14.7%	9.1%	9.1%
Infectious	23.1%	35.4%	80%	39.5%	35.3%	26.4%	9.1

ER: Emergency, OR: Operating room, WR: Ward, ST: Supratentorial craniotomy, CV: Cerebrovascular abnormalities, IT: Infratentorial Craniotomy

P value was not statistically significant for any variable.

## DISCUSSION

This study provides a critical analysis of outcomes of neurosurgical patients admitted to general ICU of a tertiary care center in Pakistan. Like other LMIC, comprehensive data on critical care outcomes of neurosurgical patients is also lacking from Pakistan. Hence, making it challenging to evaluate the effectiveness of current practices and implement quality initiatives. The current study aimed to address those gaps and identified key factors, which be the valuable contribution to the existing data from LMICs. The findings specially highlighted the patients demographics and clinical practices which would be instrumental in improving outcomes.^10^


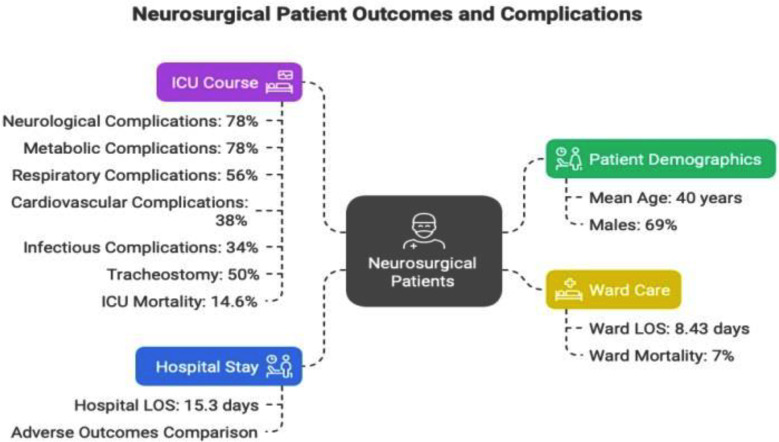


Inforgraphic Abstract : Generated by using napkin.ai Declaration of generative AI and AI-assisted technologies in the writing process The infographic abstract has been developed using napkin.ai

The key finding of our study is ICU mortality of 14.6% which is comparable to reported mortality of these patients. In fact, it is much better in comparison to outcome data of resource limited setups Acharya et al., in his retrospective review examined 813 patients from single center ICU of Nepal.^3^ Overall, mortality rate in their combined ICU was 32.8%. The difference in outcomes might be linked to type of admission, disease etiology and local demographics. In retrospective observational study authors from Nigeria shared outcomes of patients from last five years.^11^ Out of 316 neurosurgical patients, 70% (n=224) were admitted because of TBI with reported mortality of 34.6%. Similarly, ICU and overall mortality of our trauma patients was also high though it was statistically insignificant. However, they also recommended the provision of dedicated neurocritical care (NCC) for quality care of these patients. Our study patients were mostly young males, a finding that contrasts with the demographic profile observed in previous work by Broessner et a.^12^ The study involved 1,115 patients from dedicated NCC of Austria, examined survival rates and long-term functional outcomes. Their patient population was predominantly females and common reasons for ICU admissions were cerebrovascular disorders. Overall mortality was 18% for their dedicated NCC. The study also underscored the factors associated with long term functional outcomes of after discharge from the ICU. This is one of the key factor needs to be looked at for these kind of outcome based studies, especially in scenario of care for neurosurgical patients. The global neurocritical care PRINCE two survey included 1545 patient from 147 participating sites including 31 countries shared some similarities.^13^ They had predominantly male patients required ICU after subarachnoid hemmorhage. Though many of them belongs to North America, but most of them had base line comorbid conditions. Similar to our finding, admission from WR and ER were associated with poor outcomes. Considering multi center nature of trail, there was a wide variation in patient’s characteristics and provided clinical care, while certain risk factors were universal.

The impact of financial burden is significant and definitely the cost issues cannot be ignored from current discussion. A recent systematic review of nine studies (n = 2227) recommended to limit the ICU care for selected group of neurosurgical patients (Supratentorial Craniotomy) and showed related cost benefits.^14^ However, our data showed significant number of complication in patients having supratentorial craniotomy. About 39.5% of them had association with ICU acquired complications. Around 44.7% of them, required tracheostomy and in isolation had ICU related mortality of around 17.6%. Similarly there was a frequent association with systemic and neurological complications. This raises the questions about level of care for post operative patients depending upon resources. Though the cost of the ICU might be saved, but worsening of WR course and bounce back or readmission to the ICU has also been linked to higher LOS including morbidity and mortality.^15^ Therefore, the potential short term cost saving must be weighed against long term cost, poor outcomes and complications from readmission.. The common factors behind such readmission are respiratory failure and sepsis. Though we have excluded patient who requires readmission in the ICU but, patients admitted from WR in our cohort showed poor outcomes in general. The incidence of respiratory and infectious complication in them was around 80% during ICU stay. The reported mortality was also high that is 20%. The similar results has been shown by work done by Asghar A et al. In their retrospective review, they found higher association of infectious complication in patients admitted for non-surgical cause specially from WR. The association of sepsis doubled the mortality of ICU patients in this study (51%).^16^

The ICU course of our patients was associated with significant number of complications. Apart from neurological outcomes, patients also had systemic complications. Association of these complications has been linked with increased in morbidity and mortality.^17^ The geographical variation, organizational and related disparities are also linked to adverse outcomes. review Purnell et al.^18^ identified racial and ethnic minorities, rural populations, and individuals from low-middle-income backgrounds as key demographics facing health inequities that contribute to sub optimal care. The scenario might be relevant to our health care setup for which multi-center trial is the answer.

The ICU related care of neurosurgical patient also varies with the type of administrative setup, patient volume and expertise of team. Evidence shows that dedicated NCC team is associated with reduced morbidity and mortality,^19^ shorter LOS in hospitals^20^ potential cost benefits.^21^ We have combined ICU for all types of surgical patients. However, we are high volume referral center for neurosurgical cases and that may be the reason of lower mortality (14,6%) in general. The Brazilian study of similar ICU setups like what we have included 4,245 patients from 36 nationwide centers. They reported overall 28.1% of patient required ICU care for neurological disorders. Overall mortality was higher in neurosurgical cohort was 1.7 times higher in contrast to other patients (17.2% versus 10.1%).^22^ Variables like age, low GCS and severity of illness at the time of presentation were some of the independent predictors of mortality and poor outcomes. By and large recommendation is to have the provision of dedicated NCC. In one of the recent meta-analysis, included 26 observational studies, authors concluded 17% of relative risk reduction risk reduction in patient who received specialized neurocritical care.^23^

To facilitate delivery of NCC in resource-limited settings, proposed strategies should include administrative reforms including teaching and training and multidisciplinary teams collaboration.^24^ Developing work force dedicated to NCC and strategic investment in technologies should be the planning for improving intensive care outcomes of neurosurgical patients.^25^

### Limitations:

The results of this study are derived from a single tertiary center, which restricts their applicability to other institutions that may exhibit different patient demographics, clinical practices, and resource availability. Furthermore, the ICU in our study was not a dedicated NCC, potentially lacking the specialized training and advanced equipment found in such facilities. The mentioned limitations could influence patient outcomes and the reliability of the data when compared to findings from advance NCC and ultimately the overall outcomes. Moreover, we did not observe the functional outcomes and long term recovery profile of our patients that needs to be taken into consideration for future studies.

## CONCLUSION

This study offers important insights into the outcomes and complications of neurosurgical patients admitted to the ICU, revealing a high incidence of complications related to neurological, metabolic and respiratory issues. These findings emphasize the complex management required for such patients, reinforcing the need for specialized NCC. The source of admission and primary admitting diagnoses play a critical role in determining complication and related outcomes. Postoperative observation and drop in GCS were the commonest reason of ICU admission. The overall rate of complications was higher in patients coming from ward and so is the need of tracheostomy. The overall mortality was also higher in trauma patients. While the study outcomes were not statistically significant and limitations such as its single-center design, but results underscore the need for tailored interventions and resource allocation to improve care of neurosurgical patients requiring ICU stay.
